# La clínica de la subjetividad en la medicina mental española, 1870-1910

**DOI:** 10.1590/S0104-59702026000100009

**Published:** 2026-05-11

**Authors:** José Javier Plumed Domingo

**Affiliations:** i Profesor asociado, Departamento de Medicina/Universidad de Valencia. Valencia – España. j.javier.plumed@uv.es

**Keywords:** Psicopatología, Subjetividad, Medicina mental española, Psychopathology, Subjectivity, Spanish mental medicine

## Abstract

El artículo estudia los cambios en los modelos psicopatológicos establecidos en la medicina mental española entre los años 1870 y la década de 1910, utilizando como fuente principal historias clínicas y textos de referencia publicados en diversos medios en dicho periodo. Se discute cómo, simultáneamente a la introducción de la teoría de la degeneración, hubo cambios conceptuales entre los que se encuentra la incorporación de la nueva psicopatología francesa constituida a finales de siglo XIX, que permitieron avanzar en un nuevo marco de referencia teórico para captar la experiencia interna de la locura.

## Abstract (en)

This article studies the changes in the psychopathological models established in Spanish mental medicine between 1870s and 1910s. Its primary sources are clinical histories and reference texts published in various media during this period. The study discusses how the introduction of the theory of degeneration occurred with conceptual changes, including the incorporation of new French psychopathology that emerged at the end of the nineteenth century. This integration allowed for the development of a new theoretical framework to better understand the internal experience of madness.

La construcción de la psicopatología tuvo lugar en Europa a lo largo del siglo XIX y principios del siglo XX. Este proceso necesitó introducir nuevas perspectivas de análisis del sujeto; entre ellas, la inclusión de las vivencias subjetivas del paciente se ha considerado como la aportación más importante hecha a la clínica en el periodo (Berrios, 1996, p.26). Tal como comenta este autor, los cambios en la teoría psicológica, principalmente el concepto de experiencia interna desarrollado por Maine de Biran (Goldstein, 1987, p.259-261), hizo posible que los “contenidos de la conciencia” se convirtieran en un campo legítimo de investigación, aspecto que se desarrolló con una profundidad progresivamente mayor.

Varios historiadores señalan diversas fases en el proceso de construcción del discurso psicopatológico. Así, durante las primeras décadas del siglo XIX se definieron los primeros síntomas mentales a través de la obra de Pinel y, principalmente, de su discípulo Esquirol. Este último autor estableció lo que Lanteri-Laura (2000) definió como esbozo de una psicopatología, caracterizada por una tendencia a identificar una supuesta lesión con la expresión sintomática, siguiendo el método anatomopatológico de Bichat. Esta visión fue clave en la conceptualización de síntomas como la alucinación (Huertas, 2008), lo que implicaba una tendencia a la fragmentación de la experiencia mental, que muchos frenópatas consideraban como un sumatorio de fenómenos psíquicos producidos por el daño o irritación de áreas cerebrales concretas (Novella, 2018, p.50-53).

Varios cambios se produjeron en torno a la década de 1850, momento que Lanteri-Laura (2000) denominó la fase de construcción de las enfermedades mentales, tras un periodo previo en el que prevaleció un modelo unitario de locura (Berrios, Beer, 1994). Esta fase supuso un cambio de paradigma, ya que se definió tomando como base nuevos parámetros, principalmente un curso, pronóstico y clínica determinados. Esta aproximación requería un método de observación que permitiera la distinción entre cuadros clínicos claramente diferenciados entre sí. El estudio del campo psicopatológico fue protagonizado por los delirios crónicos, cuya exploración conllevó una larga discusión teórica que abarcó desde las últimas décadas del siglo XIX hasta las primeras del siglo XX. Entre los autores que protagonizaron este debate destaca Valentín Magnan, que fue uno de los grandes autores de la psiquiatría francesa tanto por la originalidad de sus propuestas como por la finura psicopatológica de sus análisis. Entre sus aportaciones destaca la descripción del delirio crónico de evolución sistemática, que se convirtió durante décadas en el modelo preponderante en la clasificación de los delirios crónicos de la escuela francesa (Álvarez, 1999, p.248-249). La característica diferencial de esta enfermedad mental, distinta de otros cuadros en los que el delirio también estaba presente, era su presencia en sujetos no afectados por la degeneración, cuyo trastorno evolucionaba a largo plazo como consecuencia de la resistencia de su mente a la irrupción mórbida, en cuatro fases bien definidas: humor delirante, delirio de persecución, delirio de grandezas y demencia como estadio terminal (Bercherie, 1986, p.100). Por su parte, la psiquiatría alemana renunció al modelo unitario de locura a través de autores como Kahlbaum y Hecker, que también sostuvieron el valor de la evolución temporal en la definición de enfermedad mental e influyeron notablemente en el trabajo de Kraepelin, principalmente con su distinción entre la demencia precoz y la psicosis maniacodepresiva, diagnósticos psiquiátricos que han llegado hasta hoy (Kendler, Jablenski, 2011).

La existencia de enfermedades mentales, definidas con estas premisas, generó la necesidad de precisar y redefinir el lenguaje psicopatológico y, con ello, construir un nuevo modo de evaluar al paciente. El último tercio del siglo XIX se ha considerado como “la edad de oro de la psicopatología”, y en este periodo destacaron dos escuelas: la más sintética, la Escuela de Santa Ana, encabezada por Magnan y seguida por Garnier, Legrain, Sérieux y Capgras; y la más sofisticada, la Escuela de la Salpêtrière, formada por Séglas, Chaslin, Cotard, Arnaud y Ballet, entre otros autores (Bercherie, 1986, p.66). Esta última etapa implicaba una diferenciación novedosa entre nosología y clínica, ya que no se trataba solo de definir un cuadro por su evolución y analizarlo para establecer un patrón clínico, sino que la tarea consistía también en distinguir entre la expresión individual de la psicopatología y el modelo general que caracterizaba un trastorno, ya que los diagnósticos no respondían a un esquema único y había toda una gama de expresiones intermedias en la clínica que el psicopatólogo debía precisar (p.102).

La mayor preocupación por la coherencia de la experiencia interna del final de siglo se ha relacionado con el cambio cultural en la naturaleza del sujeto decimonónico, fuertemente influenciado por el espiritualismo y caracterizado por su tendencia al individualismo, a desarrollar una conciencia reflexiva, y al cultivo de la interioridad (Novella, 2018, p.44). El interés por la visión integrada de la patología mental llevó al intento de explicar los elementos característicos de la experiencia delirante a partir de un hecho estructural que les daba sentido, con ejemplos paradigmáticos como el delirio de interpretación de Sérieux y Capgras (2007; original de 1909), definido en torno al núcleo de la interpretación delirante. Otros síntomas clínicos que recibieron la atención de los psicopatólogos del momento fueron tanto la experiencia predelirante, con los fenómenos elementales afectivos que la caracterizan, como los rasgos caracteriales que definen la organización psíquica del paciente psicótico. Así, Gilbert Ballet describió con detalle las alteraciones previas a la aparición del delirio en el sujeto: tristeza, timidez, inquietud, humor sombrío, vanidad y orgullo; cuya identificación requería un análisis psicopatológico más fino que el desarrollado por otros autores (Álvarez, 1999, p.251).

Este dispositivo semiológico del que hablamos quedó reflejado en las observaciones clínicas. Jean Paul Falret propuso un nuevo estilo para construirlas, de acuerdo con el cual el médico tenía que mostrar una actitud creativa y no simplemente descriptiva, y caracterizar “la individualidad enferma” del paciente en la evaluación psicopatológica. Esta transformación de la historia clínica, que se produjo en Francia desde mediados del siglo XIX, hizo que en trabajos y tesis doctorales se hiciesen más frecuentes y extensas algunas descripciones clínicas promocionadas por los maestros clínicos de finales del siglo XIX (Demazeux, 2019, p.193-195).

La historiografía ha subrayado la condición de España como país receptor de ideas psiquiátricas. La medicina mental española se desarrolló tardíamente a lo largo del siglo XIX, dada la elevada inestabilidad política y las carencias materiales del periodo. De la misma forma que otros campos de la ciencia, las ideas provenientes de otros países europeos, principalmente Francia, se introdujeron tardíamente. La influencia capital de este país en el corpus teórico de la medicina mental tuvo que ver tanto con el influjo cultural general francés en España como por las estancias de médicos en el país vecino, por interés académico o por exilios obligados por la situación política (Novella, 2016). Un cambio legislativo, la Ley de Beneficencia de 1849, estableció las bases de lo que sería la asistencia en España a lo largo del siglo. Por un lado, una red asistencial dependiente del Estado, pobremente medicalizada y regida por religiosos. Por otro lado, se constituyeron centro privados, regidos por las figuras más destacadas de la medicina mental, con vocación empresarial y donde se construyó tanto el proyecto asistencial como la elaboración teórica de la medicina mental española, con escasas aportaciones originales (Huertas, 2002). Entre las figuras que llevaron adelante estos proyectos destaca Juan Giné y Partagás, el frenópata más importante del siglo XIX en nuestro país (Diéguez, 1998). Este autor fue el fundador de una de las instituciones privadas más importantes del siglo, el manicomio de Nueva Belén. Así mismo, organizó en 1883 el primer congreso de medicina mental en España, donde participaron importantes ponentes como Magnan y Regis (Villasante, 1997), y publicó el primer tratado de psiquiatría español, el *Tratado teórico-práctico de frenopatología o estudio de las enfermedades mentales fundado en la clínica y la fisiología de los centros nerviosos* (Giné y Partagás, 1876). Su obra continuó a través del trabajo de sus discípulos más destacados, principalmente Arturo Galcerán Granés, Pedro Ribas Pujol y Antonio Rodríguez Morini. Estos frenópatas publicaron varios trabajos en la *Revista Frenopática Barcelonesa* – fundada por Giné –, primera revista científica importante editada en España. Entre las publicaciones destacan los casos clínicos, en los que se deja traslucir una teoría sobre la enfermedad mental, un modelo de sujeto y una terapéutica ajustada a esta ideología (Plumed, Rey, 2004). Hemos de destacar que, antes que elaborar un corpus teórico propio, la intención de los alienistas del último tercio del siglo XIX fue establecer las bases para institucionalizar la medicina mental española y el organicismo al que muchos de ellos se acogieron estaba estrechamente ligado a la voluntad de dar un estatus científico a la nueva especialidad, que le permitiera situarse al nivel de otras especialidades médicas (Huertas, 2002).

La introducción de la teoría de la degeneración en nuestro país comenzó en torno a la década de 1880. En España su modelo tuvo una importancia notable dentro del proceso de asentamiento de la especialidad, principalmente a través de su influencia en los peritajes médicos en los tribunales de justicia, así como fue un instrumento esencial a la hora de teorizar programas de higiene mental. Sin embargo, como también se ha señalado, su influencia en la clínica fue muy significativa (Campos, Martínez Pérez, Huertas, 2001; Campos, 1999a; Plumed, Rey, 2001). En la bibliografía se ha concedido menos atención a la introducción simultánea a esta doctrina de la nueva psicopatología y cómo condicionó, junto a otros factores, el estudio de la subjetividad de la locura en España.

En este trabajo se estudian los modelos psicopatológicos que presidieron el debate teórico en la psiquiatría española entre la década de 1870, momento en que se da un impulso a la producción científica y se asienta la medicina mental, principalmente a través de los autores de la escuela catalana (Rey et al., 2006, p.26), y 1910, década de transición previa a la aparición de los psiquiatras que conformarán la escuela de Madrid, origen de la modernidad en la psiquiatría española, y que facilitaron la introducción del psicoanálisis en la conceptualización de la enfermedad mental. El material utilizado para el trabajo está constituido, principalmente, por historias clínicas publicadas en artículos de revistas especializadas relevantes para el periodo estudiado, localizadas a partir de los repertorios existentes (Rey et al., 2006), así como los tratados publicados sobre medicina mental en dicho periodo de tiempo y libros donde las historias clínicas ocupan un espacio significativo.

## La clínica psiquiátrica en la escuela de Giné y Partagás

Se han escrito distintos trabajos sobre los fundamentos teóricos de la escuela de Giné y Partagás (Huertas 2002; Diéguez, 1998), que vienen claramente representados en su *Tratado* (Giné y Partagás, 1876), donde expuso sus principios teóricos sobre la enfermedad mental y la psicopatología. Independientemente de que la obra de este autor presente aspectos psicologicistas, visible en la influencia de autores como Joseph Guislain (Diéguez, 1998) y que se hizo notoria en trabajos más tardíos como su novela *Misterios de la locura. Novela científica* (Giné y Partágas, 1890), en la cual se refleja un interés y preocupación evidentes por la experiencia subjetiva del individuo (Huertas, 2010), la perspectiva de estudio de la enfermedad mental para Giné es, principalmente, organicista. Múltiples aspectos de su obra remiten a este hecho. A modo de ejemplo significativo, casi cien páginas del *Tratado* están dedicadas a las estructuras del sistema nervioso central, que están descritas como la parte esencial del conocimiento de la frenopatía. Simultáneamente, en la sección dedicada al estudio de los cuadros clínicos, los supuestos anatomopatológicos que los definen ocupan un lugar preponderante. Así, el capítulo dedicado a la melancolía muestra una simbiosis entre la clasificación promulgada por Guislain y un planteamiento radicalmente organicista. Escribió el autor:

La interpretación patogenética de este cuadro sintomatológico se deduce de las nociones que tenemos acerca de las propiedades fisiológicas específicas de los elementos anatómicos del cerebro y de sus íntimas conexiones anatómicas y dinámicas ... la alteración primitiva recae sobre las células afectivas y ellas son las únicas perturbadas al principio de la enfermedad (Giné y Partagás, 1876, p.394-395).

Este fundamento biologicista tuvo implicaciones evidentes en la concepción psicopatológica de Giné y Partagás. En este sentido, mostraba una tendencia a la visión fragmentaria de los síntomas, fundamentados en un modelo anatomoclínico que asimilaba la frenopatía a la neurología. Un ejemplo paradigmático reside en el capítulo dedicado a la alucinación. Hace referencia a Esquirol, autor que sostenía un modelo anatomoclínico del síntoma (Huertas, 2008) y a los modelos de Virchow y Luys, que mostraban un somaticismo aún más radical. “Luys, consecuente con su doctrina anatomofisiológica de los centros nerviosos, localiza las alucinaciones en los centros de los tálamos ópticos” (Giné y Partagás, 1876, p.162). Aquí Giné sugería que un síntoma no se produce por una afectación global de la mente del sujeto, sino como consecuencia de una disfunción de un área específica que produce un trastorno parcial de la experiencia normal. Con la misma línea argumental, Galcerán Granés, principal discípulo de Giné y Partagás y figura emblemática de la psiquiatría catalana de entresiglos, hablando sobre el trastorno de las asociaciones de ideas, consagraba un modelo de yo en el que las distintas funciones presentaban un desarrollo independiente, asociado a una determinada área cerebral, con lo que definía una perspectiva localizacionista que desarrollaría en su obra posterior: “Nuestros lectores recordarán, por anteriores trabajos de esta Revista, que la memoria, la voluntad, la volición, enferman independientemente de cualquier otra propiedad o función cerebral” (Galcerán Granés, 1883, p.282).

Estos planteamientos defendían un modelo de sujeto normal y patológico claramente diferenciado por la lesión que originaba el cuadro, cuya identificación y tratamiento sería el objetivo del frenópata. Implícitamente, la teoría de la locura propuesta por Giné mostraba un interés limitado por la subjetividad del loco y tendía a fundamentar el análisis psicopatológico en la conducta del paciente y su discurso explícito. Esta búsqueda de la objetividad en la definición del síntoma perseguía construir una ciencia de la mente acorde con el método empírico y ser fiel al modelo teórico que subyacía a la visión de los autores organicistas (Goldstein, 1987, p.247). Resulta congruente con esta perspectiva que los matices diferenciales de la experiencia de los pacientes tuviesen un peso muy relativo a la hora de establecer tratamientos y diagnósticos.

La publicación de historias clínicas por autores españoles en la prensa médica fue relativamente esporádica entre las décadas de 1850 y 1870, hasta que aumentó el número de trabajos a partir de los años 1880. El escenario más importante donde fueron expuestos fue la *Revista Frenopática Barcelonesa*, publicada entre 1881 y 1884, donde los discípulos de Giné y Partagás publicaron un total de 48 historias clínicas (Plumed, Rey, 2004), que mostraban el modelo teórico propio de la escuela a la que pertenecían. En su estructura, además de consignar el temperamento y la constitución del paciente, de forma esquemática y rutinaria, indicaban factores etiológicos, centrados en las causas ocasionales de enfermedad. Aquí hemos de reseñar que el peso para el sujeto del hecho desencadenante no queda reflejado en las historias como algo significativo del devenir posterior de la clínica, sino como un fenómeno accesorio.

En lo que respecta a la descripción psicopatológica, los autores exponían tanto los síntomas físicos objetivables como los psicológicos. Dentro de los físicos señalaban signos generales, como el pulso, palidez y frecuencia respiratoria, que reflejaban de manera objetiva el estado mental del sujeto. Como ejemplo, en un caso titulado como “manía general aguda con predominio del delirio suicida” se describía así el estado del paciente:

Los surcos nasogenianos, naso-labiales, palpebrales y el mento-labial en extremo pronunciados, la respiración fatigosa y acelerada, la frecuencia del pulso notablemente desplegado, la inquietud y sobresalto de que estaba poseído y otros síntomas objetivos muy marcados, eran la más fiel expresión del terrible delirio que le atormentaba (Ribas Pujol, 1882b, p.337).

Respecto a los síntomas mentales, en la mayoría de los casos aparecían descritos sin detalles que ayudasen a diferenciar su expresión particular en el paciente estudiado. Así, en un caso de manía, la descripción psicopatológica de Rodríguez Morini, futuro director de la *Revista Frenopática Española* y director del Manicomio de San Baudilio de Llobregat, se resumía así: “El delirio es más tranquilo, hay más coherencia; las alucinaciones auditivas no son tan intensas; la afectividad renace, aunque lentamente” (Rodríguez Morini, 1885, p.80). Otros, por el contrario, mostraban una elaboración algo más detallada, donde se evidenciaba un intento de incorporar el contenido particular que el síntoma adoptaba en el paciente, como vemos en este ejemplo:

Las personas más adictas eran sus más encarnizados enemigos, tales como su madre, sus hermanos y, en especial, un tío suyo; razonando que los primeros pretendían quitarle de su presencia, y que éste último deseaba darle muerte y ahorrarse de pagar una crecida cantidad que había heredado el enfermo y con la cual pensaba eximirse del servicio de las armas (Ribas Pujol, 1882a, p.174-175).

El testimonio directo del enfermo aparecía en un número limitado de historias y se utilizaba, en buena medida, para demostrar la precisión de las observaciones hechas por el especialista. Como ejemplo, el paciente del caso citado anteriormente intentó simular una mejoría, que fue detectada en el seguimiento clínico a pesar de los esfuerzos teatrales del enfermo, que se sometía sin queja al régimen de la institución y era capaz de escribir una carta a su madre ocultando su verdadero estado: “Mi querida madre… para que vengáis y yo pueda, así , salir de esta casa que tanto me pesa desde que sé que en breves días he de encontrarme otra vez en el seno de mi familia libre e independiente de mis torpes manías…” (Ribas Pujol, 1882a, p.177).

El diagnóstico se basaba en el esquema nosológico procedente de Pinel y Esquirol, y sus categorías diagnósticas de manía, melancolía y demencia. En general la dimensión temporal no estaba adecuadamente integrada, y las distintas manifestaciones clínicas se consideraban complicaciones del trastorno inicial, como indicaban los títulos de varios trabajos. Como ejemplo, en un caso de Ribas Pujol (1881), se describía un episodio de melancolía que se complicaba con manía vanidosa y se acaba curando por los beneficios del ingreso. Esta carencia se manifestaba también en el relato, de forma que la sucesión de síntomas no obedecía a una narrativa coherente que describiese la vivencia interna del paciente, sino que se exponía de forma relativamente fragmentaria, de modo congruente con los planteamientos teóricos que estamos describiendo.

La perspectiva descrita de la escuela de Giné y Partagás dio paso a un cambio significativo en la concepción del paciente, vehiculizado por la introducción de la teoría de la degeneración en España. Se ha estudiado la influencia de esta teoría en la psiquiatría española, desde su vertiente clínica, profesional y social (Campos, Martínez Pérez, Huertas, 2001). Sin embargo, otro aspecto a considerar es el papel de algunos de los autores más destacados de esta escuela en la construcción de la subjetividad del loco, en la medida en que ayudó a introducir elementos clínicos novedosos para mostrar una visión más precisa y compleja de la experiencia del enfermar y el proceso de transformación que supone para el individuo.

## La detección del estigma psíquico

Valentín Magnan fue el autor más traducido de la medicina mental española del siglo XIX (Rey, 1981). Su modelo teórico tuvo una aceptación importante y hubo una preferencia en su adopción por encima del trabajo de Morel. Entre sus aportaciones, una de las más notables fue la noción de estigma físico, una afectación morfológica del paciente donde se objetivaba el proceso degenerativo que producía la enfermedad mental (Campos, 1999a). Por otra parte, Valentín Magnan y Maurice Legrain (1895) postularon la existencia del estigma psíquico, una marca afectiva o conductual que indicaba la existencia de una lesión psíquica, cuyo avance iría creando el camino para la locura. El acúmulo de estigmas psíquicos definía un perfil psicológico que sería superponible al de la personalidad premórbida y concebido como una forma atenuada de locura (Berrios, 1993), con lo que el caduco concepto de temperamento se dejaba de lado para llegar a un modelo patológico de organización del individuo cuya transformación conducía a la enfermedad mental. De esta forma, la persona se definía por una historia que, aunque tuviese un origen biológico de fondo, se modificaba en función de un ambiente social que daba sentido al desenlace funesto, la locura. Hemos de recordar que un elemento fundamental en la constitución de la subjetividad del individuo es su continuidad temporal. Por ello, el yo no puede entenderse separado de sus vivencias y está constituido por ellas, creando una narrativa que da un marco de inteligibilidad a la existencia (Zahavi, 2005, p.113-132). Por ello, la concepción de una clínica psiquiátrica ligada a las experiencias vitales cabría entenderla como un paso importante en la construcción de un modelo de subjetividad.

Hemos de recordar que estos desarrollos conceptuales se produjeron en un contexto cultural concreto, en el que las literaturas realista y naturalista llegaron a su apogeo. Como señala Pick (1989), la influencia de la mentalidad positivista – convirtiendo el arte en un estudio de observación donde se pudiese dar cuenta de la naturaleza real de la experiencia humana teniendo en cuenta el conocimiento científico – fue determinante en la obra de autores naturalistas como Emile Zola, ampliamente discutido en la época. En este sentido, Huertas (1984) subraya la influencia en este autor de la obra de Morel y Magnan y su intento, a través de la literatura, de buscar una verdad basada en la ciencia que fuese aplicable al conocimiento psicológico. Como comenta Campos (2012, p.53), las descripciones aparentemente científicas que aparecían en los textos de escritores realistas tenían un tono literario obvio a pesar de su voluntad de ser objetivas, de modo que literatura y ciencia se imbricaban en una mixtura de influencia mutua difícil de separar. Este entorno cultural afectó a la concepción de las narrativas sobre la locura y contribuyó a que veamos un cambio brusco en la estructura y forma de las historias clínicas influidas por el degeneracionismo en la década de 1880. De manera mucho más acentuada que en los casos publicados de Nueva Belén, existía una voluntad por parte de estos autores de consignar una predisposición caracterial, un intento de filiar la influencia de la sociedad en la clínica, desde la familia a otros entornos cercanos, y una historia que recogiese el devenir de las interacciones vitales y su relación con el trastorno mental.

La historiografía ha analizado la importancia de los casos judiciales en España en el proceso de institucionalización de la especialidad, dado que permitieron a los frenópatas hacer patente su papel de expertos en campos ajenos a la práctica médica (Campos, Martínez Pérez, Huertas, 2001; Campos, 1999b, 2003). Al igual que ocurrió en Francia, las actuaciones de los psiquiatras como peritos en los tribunales de justicia se convertiría en un instrumento de gran importancia para divulgar ante la sociedad un modelo medicalizado de criminal basado en la teoría de la degeneración, dada la amplia proyección social y mediática que tuvieron este tipo de casos. Uno de los ejemplos más representativos fue el caso Garayo (Esquerdo y Zaragoza, 1881). Este criminal, entre 1870 y 1879, asesinó y violó al menos a seis mujeres, de edades comprendidas entre 13 y 55 años, tragedia que se convirtió en un suceso muy popular en la España de la época. Aunque Garayo fue finalmente ejecutado, el papel de los médicos que participaron en el peritaje tuvo una gran importancia. La finalidad estratégica de este juicio, para los médicos mentalistas, fue, por un lado, convencer a los jueces de la importancia del conocimiento psiquiátrico para determinar la imputabilidad del reo y, por otro, influir tanto en la confección de un nuevo código penal como en la actitud de la opinión pública, generando una visión favorable sobre la importancia del conocimiento experto del médico (Álvarez Uría, 1983, p.202-203). El peritaje de José María Esquerdo – fundador del manicomio de Carabanchel Alto, político progresista, que llegó a ser diputado por el Partido Republicano, gran defensor de la importancia de la nueva ciencia frenopática y, sin duda, la figura más emblemática de uno de los núcleos de actividad más importantes de la psiquiatría madrileña de finales del siglo XIX – fue ampliamente difundido en la prensa (Huertas, 2003). Aunque daba gran importancia para el diagnóstico a la descripción de los estigmas físicos del enfermo, también eran determinantes para clarificar la clínica los estigmas psíquicos que, lejos de ser anecdóticos, anunciaban el cuadro y contenían aspectos nucleares del delirio desarrollado: el descontrol del impulso genésico y la incapacidad de reconocer sentimientos por el otro. Así mismo, comentaba el autor con profusión hechos puntuales de carácter disruptivo (impulsividad, violencia inusitada) que llevaron al sujeto al crimen y que sólo su degeneración mental podía justificar. Escribió Esquerdo y Zaragoza (1881, p.153; destacados del autor):

Fue siempre laborioso, interesado y glotón, egoísta, independiente, taciturno y apagado, pero ‘se animaba en presencia de las mujeres, con las cuales se permitia alguna chunga’. No amaba a sus hijos ni a sus mujeres, a excepción de la primera. Fue siempre para todos ellos tacaño y enemigo de hacer sacrificio alguno.

Esta visión del peritaje, que combinaba la objetividad de la descripción física del sujeto con un esfuerzo por aproximarse a su experiencia interna, fue también seguida en diversos casos judiciales por un discípulo de Esquerdo, José María Escuder, siendo el más significativo el publicitado *El caso Morillo* (Campos, 2012). Manuel Morillo, médico de profesión, asesinó en 1883 a la madre de su novia en Madrid. El caso fue difundido ampliamente por la prensa, con lo que tuvo una considerable resonancia social. Escuder (1895, p.31-85) desarrolló ampliamente el esquema de su maestro y analizó la historia desde una perspectiva compleja. En su descripción clínica incluía fragmentos de la tesis doctoral del paciente, textos autobiográficos y una descripción pormenorizada tanto de los acontecimientos vitales que sufrió, principalmente la pasión amorosa, como la manera particular de experimentarlos y la forma en que dichas vivencias le condujeron al delirio. Aquí vemos cómo el análisis detallado de la subjetividad del enfermo, considerada desde su biografía, permitía un diagnóstico preciso. Víctima de un delirio religioso, relacionaba su crianza con un sacerdote catedrático de educación y moral, cuya protección vio interrumpida por una muerte temprana, con el contenido de su delirio.

Desde una perspectiva similar, en otro caso denominado “el veterinario de Sueca”*,* Escuder (1895, p.5-30), a partir del asesinato que un veterinario cometió con ayuda de un cómplice, utilizaba el material clínico del enfermo, una carta autobiográfica, así como una entrevista hecha al sujeto en prisión, con la que construía un relato donde los estigmas psíquicos del reo (“susceptible, iracundo y cabezudo”), justificaban las actitudes y comportamientos que había desarrollado previamente a la aparición de la locura: “Fue alcalde y capitán de una partida, en la que se distinguía por su crueldad e impulsiva decisión alternada por cobardías repentinas y miedo a los muertos, por cuyas almas rezaba frecuentes rosarios” (Escuder, 1895, p.11). Llama la atención el detalle en la descripción de las vivencias que llevaban al sujeto a cometer el crimen, donde encontramos un intento decidido de profundizar en la naturaleza íntima del protagonista del caso, más allá del comportamiento aparente. Escuder intercaló fragmentos de diálogo para dar viveza y autenticidad a la descripción, así como se esforzó en justificar psicológicamente en su relato los dilemas del paciente (la elección como cómplice del crimen a un joven de 19 años que descubrió en el matadero; los tormentos y contradicciones del asesino antes del crimen). La utilización de recursos dramáticos para dar fuerza y credibilidad a su historia es notoria, mediante la expresividad de sus comentarios y descripciones. No podemos olvidar que hubo géneros populares de perfil realista, como la novela por entregas y los folletines publicados en la prensa diaria, que influyeron claramente en la elaboración de casos aparecidos en la prensa médica, como en el ya citado caso de Garayo (Campos, 2012, p.38-39). Vemos cómo la narrativa utilizada para describir el caso presenta un psicologicismo cargado de dramatismo y recursos literarios llamados a emocionar al lector, trascendiendo la mera descripción médica. De la misma forma, hacía patente cómo el relato forense de la locura solo podía construirse desde la evidencia que genera el conocimiento tanto de la naturaleza de la enfermedad mental como de la capacidad de penetrar en la mente humana tomando, por ello, como campo de estudio la vida interior del sujeto.

Años más tarde, Bravo y Moreno y Roig y Boet (1908), médicos forenses, publicaron un caso en el que juzgaban al autor de un atentado terrorista. En su evaluación resaltaba su voluntad de ofrecer una historia del sujeto que recogiera no solo sus antecedentes genéticos, sino sus circunstancias de vida, comportamiento y vivencias infantiles y adolescentes. En la historia clínica subrayaban los autores la reacción emocional anómala del paciente en su niñez (“no conoció el llanto ni cuando lo destetaron”, p.230), su dificultad para relacionarse con compañeros (“durante un año que estuvo en la escuela fue la chacota y befa de los demás muchachos, los cuales lo llamaban la boba”, p.231). Y, dadas sus limitaciones a la hora de encontrar un trabajo, describían cómo se vio arrastrado a una vida de vagabundeo cuyo fundamento radicaba en sus estigmas psíquicos y que justificaba la clínica que le arrastró al crimen:

Escapándose de su casa hace cosa de tres años, durante los que se dedicó a la vagabundez, adquiriendo relaciones con gentes de mal vivir y frecuentando el trato de perdidos, y encontrándose sin recursos, se entregaba a las ocupaciones que menos se compaginan con su origen, alistándose de marinero unas veces, otras pidiendo; más incapaz de perseverar en un oficio honroso o en una profesión honesta por efecto de su inestabilidad mental (Bravo y Moreno, Roig y Boet, 1908, p.231).

La incorporación del carácter del individuo y su biografía como parte integral del estudio psiquiátrico aparece también recogida en los casos clínicos de la prensa médica general donde, como hemos visto en los ejemplos anteriores, hay un esfuerzo significativo por recoger la influencia de las circunstancias vitales y su relación con los síntomas de la enfermedad establecida. No podemos olvidar que el degeneracionismo, aunque excluía interpretaciones puramente psicológicas de los fenómenos mentales, aceptaba la mutua influencia entre aspectos psíquicos y fisiológicos, lo que facilitó que los médicos mentalistas continuasen atribuyendo un valor patogénico a las causas morales (Dowbiggin, 1991, p.130).

Es de interés resaltar que la mayoría de los casos publicados fueron atendidos en centros hospitalarios medicalizados, como las consultas externas del Hospital de la Princesa (Fernández Sanz, 1908), centros privados de pago como el Instituto Frenopático de Les Corts, dirigido por Luis Dolsa y Ramon (1879), o en centros de atención a grupos sociales relativamente elitistas, como el Sanatorio Psiquiátrico San José de Cienpozuelos, donde se trataban militares, en varios de los casos de cierto rango, como capitanes y otros mandos (Fernández Victorio y Cociña, 1908). Aunque no se suele indicar en la mayoría de las historias si se trataba de enfermos de pago, encontramos datos que hablan a favor de un estatus socioeconómico alto de un número significativo de los pacientes descritos en alusiones concretas. Por ejemplo, en un enfermo atendido en el manicomio de Zaragoza se comenta que el padre es “cargo importante en una gran compañía” (Gimeno Riera, 1909, p.336). Además de la esperable mejor atención a los enfermos de pago frente a aquellos hacinados en instituciones pobremente dotadas, probablemente exista un sesgo en las historias hacia pacientes de mejor nivel educativo y capaces de comunicarse de forma más adecuada, como muestra el alto número de enfermos con un nivel de estudios medio o alto. Así, en un caso de locura alcohólica (Ots y Esquerdo, 1893a) se describe la historia de un opositor brillante y en varios casos de locura persecutoria (Ots y Esquerdo, 1893c), los ejemplos clínicos provienen de sujetos con una formación alta. Otra cuestión importante era la pobreza de las historias clínicas de los pacientes crónicos pensionados, que no ofrecían la posibilidad de ser publicadas dada la limitación de datos. Rodríguez Morini (1904), en un caso de demencia precoz catatónica ingresada en el Manicomio de San Baudilio, comentaba del paciente que “es uno de tantos infelices que llegan al establecimiento por cuenta de las Diputaciones Provinciales, sin conmemorativo patológico, y en los cuales se hace casi imposible reconstruir la historia clínica” (p.345).

Como ejemplo representativo, en el caso ya mencionado de Dolsa y Ramón (1879) se estudiaba a un paciente diagnosticado de monomanía desde una perspectiva degeneracionista. Al describirlo, el autor subrayaba los estigmas psíquicos del enfermo, generados como consecuencia tanto de la herencia como de un contexto educacional y social que le influía en una dirección irreversible: “Entonces es cuando vemos que aquel que cuidaba de sí, tan solo movido por ese sentimiento que nos obliga a adornar nuestro cuerpo o a cultivar nuestra inteligencia, a fin de ser más agradables a nuestros semejantes, y el loco convertido en un Don Juan o en otro Galileo, es un mismo individuo” (p.467).

Otro autor, Vicente Ots y Esquerdo (1892), médico frenópata del Manicomio de Carabanchel bajo la dirección de José María Esquerdo, estudió la locura religiosa ilustrándola con varios casos clínicos que seguían el modelo ya indicado. Uno de ellos se trataba de un adolescente de 19 años, sin antecedentes de herencia vesánica, cuya locura venía justificada por sus circunstancias de vida. Dada su condición de huérfano, tuvo que estar a cargo de un rígido tutor que veía con malestar sus fugas de casa y su comportamiento desajustado. Por ello, se decidió a ingresarlo en un Colegio Correccional del Mediodía de Francia, donde, como efecto de la vida ascética, empezó a desarrollar ideas delirantes religiosas.

Esta tendencia a considerar cómo los acontecimientos vitales que sufre condicionan a cada individuo de una manera particular se mantuvo con mayor o menor énfasis por parte de los autores más representativos. Enrique Fernández Sanz (1908), uno de los introductores del psicoanálisis en España y futuro director del Manicomio de Leganés, comentaba un caso de demencia precoz catatónica recordando los antecedentes vesánicos y la biografía del sujeto. Tras comentar las circunstancias del parto y los hitos del desarrollo infantil (lactancia, dentición, fecha de la marcha) exponía el brillante desarrollo académico del protagonista del caso, junto con estigmas psíquicos que constituían el germen de su trastorno mental: “Desde muy niño su carácter era excesivamente serio, impropio de su edad; rehuyendo los juegos infantiles, se encerraba para devorar cuantos libros tenía a su alcance y para llenar de números hojas y hojas de papel” (p.286). Salió de la escuela para trabajar en el campo y, dado que no encontraba salida para su afición literaria, fue a la ciudad para trabajar en una oficina de recaudación de impuestos. “Allí tenía excesivo trabajo, pero no sólo lo despachaba a completa satisfacción de sus superiores, sino que, una vez cumplida su tarea, iba a la escuela nocturna a ayudar al profesor y todavía trabajaba después solitariamente” (p.286). En este caso, señalaba Fernández Sanz cómo la disarmonía de sus capacidades, recordando el concepto clave de Magnan, las duras exigencias de vida del medio social donde vivía, el contraste entre sus expectativas de vida y la dureza de los trabajos que necesitaba ejercer para sobrevivir, fue presionando su yo hasta quebrarlo.

## El refinamiento de la semiología psiquiátrica

Un aspecto teórico extensamente discutido en la literatura médica del último tercio del siglo XIX fue la distinción entre razón y locura. Históricamente se ha considerado uno de los temas cruciales de la psicopatología y la perspectiva con que se estudió tuvo consecuencias en muy diversos ámbitos, tanto asistenciales como legales. La entidad nosológica más representativa de la primera mitad del siglo que clasificaba los cuadros situados en la frontera de la locura fue la monomanía, cuya introducción y asimilación generó enconados debates hasta la década de los años 1860 (Martínez Pérez, 1995). Esta enfermedad partía en su concepción de una discutible división de las funciones psíquicas en áreas independientes y, como se ha debatido en la literatura, tuvo un papel clave en la institucionalización profesional de la psiquiatría en nuestro país (Campos, Martínez Pérez, Huertas, 2001). Si bien se ha propuesto que la sustitución del caduco concepto de monomanía por la teoría de la degeneración se produjo en base a la objetivación que permitía el estigma físico, con lo que el papel experto del frenópata ante los tribunales de justicia quedaba reforzado (p.84-103), la “desaparición” del diagnóstico también estimuló el debate sobre las formas intermedias de locura en la clínica. Como plantean Álvarez, Colina y Esteban (2011, p.XX), “la semiología clínica se construye sobre la acentuación de las diferencias entre lo normal y lo patológico, la cordura y la locura”. En este sentido, el interés por el problema facilitó que se importase la discusión nosológica francesa de finales de siglo, que prestó una atención especial a esta cuestión. Autores como Magnan o Séglas, aunque estuviesen influidos por la teoría de la degeneración, enriquecieron la mirada psicopatológica con modelos teóricos complejos sobre las formas clínicas fronterizas. Evidentemente, esta cuestión tenía una enorme significación tanto desde la perspectiva clínica como forense y requería de un análisis más fino, ya que el diagnóstico diferencial entre las distintas enfermedades mentales se basaba en el estudio de la expresión de los síntomas, la relación entre ellos y su impacto en el individuo.

En la psiquiatría española la discusión teórica sobre las locuras parciales fue un tema recurrente en la literatura médica. Un ejemplo ilustrativo lo tenemos en el caso ya citado de monomanía de Dolsa y Ramón (1879), escrito en un momento en que el término y el concepto habían quedado desfasados. Aunque empezaba citando la definición de Esquirol, el desarrollo del caso no seguía el modelo teórico de este autor y mostraba una clara orientación degeneracionista. Insistía en tres aspectos centrales: presencia de delirio y alucinaciones centradas en un aspecto concreto de la realidad, comportamiento aparentemente normal y gradación de los aspectos patológicos desde el egoísmo o vanidad hasta los síntomas evidenciables solo desde el conocimiento preciso de la experiencia del paciente, con lo que entroncaba directamente con el problema de las locuras parciales. Otra entidad nosológica que intentó definir estas formas patológicas fue la locura lúcida. Construida por Ulysse Trélat (1861), describía a enfermos con una razón aparentemente conservada, pero que presentaban síntomas mentales indicativos de delirio que solo una mirada clínica capaz de profundizar en la experiencia del paciente permitía reconocer. Citando al autor, “su falta de razón sólo se conoce desde el interior y no se muestra afuera” (p.12). Ots y Esquerdo (1903a) relacionaba la locura lúcida con la monomanía y señalaba la importancia clínica de los enfermos que podían atender sus ocupaciones y tratar a sus contactos sociales con perfecta lucidez, pero en la intimidad de la casa y ante la mirada atenta del mentalista mostraban “prevenciones o preocupaciones insanas, temeridades insensatas, aversiones injustificadas, escrúpulos monjiles y temores quiméricos” (p.8).

El delirio crónico de evolución sistemática fue descrito por Valentín Magnan en 1886 y tuvo un notable éxito en la medicina mental española (Magnan, 1892). Aunque desde principios del siglo XX la demencia precoz de Kraepelin fue intensamente discutida, la forma paranoide de la demencia precoz nunca llegó a sustituirlo durante las dos primeras décadas del siglo XX y fue fuertemente criticada en favor de la entidad de Magnan y los cuadros de la escuela de la Salpetrière, incluyendo el delirio de interpretación de Serieux y Capgras (Plumed, 2008, p.439-441). Es comprensible que los modelos de la escuela francesa triunfasen frente a la perspectiva externalista y simplificadora de Kraepelin, ya que hasta ahora se ha expuesto cómo los principios psicopatológicos que sustentaban su nosología, basados en un interés notorio por la experiencia subjetiva, fueron bien recibidos en España desde décadas anteriores a la introducción de la perspectiva del autor alemán

El delirio crónico también contribuyó a llenar el hueco de las antiguas monomanías, como se indicó en diversos trabajos. En la *Guía del diagnóstico de las enfermedades mentales, con nociones sobre la terapéutica deontología y medicina legal frenopáticas*, de Martínez Valverde (1900), encontramos una referencia a las locuras parciales crónicas sistematizadas. Tomando una referencia textual de Magnan y Serieux, las define el autor como

delirantes, no dejan de presentar el singular fenómeno de la integridad más o menos aparente de las facultades silogísticas; son locos sin duda, pero locos que pueden vivir largo tiempo la vida común, que saben discutir, escribir memorias justificativas y defender sus convicciones con bastante destreza, a veces, para convencer a los que les rodean (Martínez Valverde, 1900, p.187-188).

Para el autor, este diagnóstico incluía a las antiguas monomanías de Esquirol, y las sucesivas fases de la enfermedad serían locuras parciales que formaban parte de la misma dolencia, como fases del cuadro evolutivo. En décadas posteriores abundantes trabajos y casos clínicos publicados continuaron discutiendo esta cuestión. Como ejemplo, Antonio Fernández Victorio y Cociña (1907), médico militar que fue catedrático de patología de la Universidad de Manila y director del sanatorio psiquiátrico San José de Cienpozuelos, presentó dos casos de internos psiquiátricos definidos como locos disimuladores, que asociaba al diagnóstico de locura consciente y que estaban caracterizados por una pérdida de la razón solo parcial. La clínica delirante dejaba de hacerse aparente en el ingreso y solo tras meses de seguimiento detallado se encontraban escritos que delataban el delirio y alucinaciones, que confesaban tras un intenso interrogatorio. El autor consideró como diagnóstico probable el delirio crónico de evolución sistemática, aunque el curso evolutivo no respondiese rígidamente al modelo de Magnan y se basaba en la descripción de los pacientes que, en un momento dado, accedían a comunicar al psiquiatra sus experiencias.

Hemos de recordar que la dimensión temporal y su introducción en el diagnóstico fue un cambio importante introducido en este periodo (Lanteri-Laura, 2000). Esta perspectiva subrayaba la importancia del estadio inicial de la enfermedad, cuando la posibilidad de reconocer señales inespecíficas no era posible desde una mirada centrada en signos patognomónicos, sino en la interpretación adecuada de los síntomas afectivos y volitivos. Para conseguir este objetivo era necesario definir la enfermedad desde su comienzo hasta el establecimiento de sus manifestaciones más aparatosas, distinguiendo adecuadamente sus cambios en el tiempo. Como acertadamente analizan Álvarez, Colina y Esteban (2011, p.XIX), “la clínica de la instantaneidad y la sincronía dio paso a una mirada historicista, evolutiva y diacrónica, cuando los fenómenos morbosos más toscos se pulieron hasta conformar una clínica de lo sutil y lo elemental”.

En las últimas décadas del siglo XIX y principios del XX, como señala Álvarez (1999), la psicopatología descriptiva, a través de la obra de Magnan y la escuela de La Salpetrière (Cotard, Arnaud, Régis, Ball, Gilbert Ballet, Séglas), adquirió su máximo esplendor. El reduccionismo de autores previos, preocupados en distinguir el signo de la enfermedad de la forma más objetiva posible, y relacionándola con un posible correlato neurobiológico, quedó en un segundo plano, y fue mayor el interés en conocer aspectos emocionales del sujeto que resultasen más o menos accesibles a la observación directa. Esta perspectiva llevaba a lo que autores como Sèglas llamaron “el análisis psicológico”, es decir, la contextualización de los síntomas dentro de la estructura de la psicología patológica de cada individuo, para ponerla a su vez en relación con estructuras diagnósticas bien delimitadas (Álvarez, Colina, Esteban, 2012, p.XII). De la misma forma, adquirieron importancia los fenómenos clínicos más sutiles, que no se reducían a la clínica delirante, alucinatoria y conductual más aparente. Para poder establecer este cambio se hizo necesaria una visión más integrada del psiquismo y, con ello, un diagnóstico que no se limitase a la identificación de síntomas patognomónicos. Como plantean Álvarez, Colina y Esteban (2011, p.XXIII), con la nueva psicopatología se perdía la especificidad del signo aislado, y la valoración nosológica solo podía hacerse a partir de la experiencia del paciente. Esta visión integrada conducía a la centralidad del equilibrio de las funciones psíquicas en la valoración psicopatológica, que se diferenciaba de las visiones más objetivantes y fragmentarias ya descritas. A pesar del organicismo de los autores degeneracionistas, el valor concedido en las historias clínicas al mundo interior del sujeto y la importancia que reciben la integridad y equilibrio del yo como aspectos centrales de la descripción psicopatológica hace difícil obviar la influencia del espiritualismo en estos autores, que así contrarrestaban la tendencia a la fragmentación y reducción del psiquismo propias de la ciencia moderna (Novella, 2013b, p.11). En este sentido, es importante recordar que existía una larga tradición de aproximaciones teóricas que subrayaban la importancia de la noción de equilibrio psíquico. Ya en la psicopatología de Pinel vimos que la locura se producía por un desequilibrio de las pasiones, y el modelo de monomanía de Esquirol radicaba en una descoordinación entre las partes afectiva, volitiva y cognitiva del sujeto. La visión novedosa de esta cuestión vino a través de la noción de disarmonía, una aportación central de Valentín Magnan, que definía la locura como un desequilibrio particular, más profundo, de las funciones psíquicas. En palabras de Magnan y Legrain (1895, p.100), “la locura ... consiste en una ausencia de armonía entre las diversas funciones, una ausencia de sinergia entre los diferentes centros que cooperan”.

Un estudio adecuado de la perspectiva del enfermo implicaba la necesidad de recoger y evaluar aspectos sintomáticos que no habían sido puestos en relieve en modelos psicopatológicos anteriores, como automatismos y fenómenos impulsivos. Como ejemplo de este interés, Martínez Valverde recogió el concepto de impulsión en su libro citando a Séglas y definía el impulso irresistible para el yo teniendo en cuenta que, en estado normal, “establece el equilibrio entre la sensación que excita, el deseo de ejecutar aquel y la realización del mismo”, y, cuando se pierde este equilibrio, “se encuentran reunidas las circunstancias necesarias para la aparición del síntoma que estudiamos” (Martínez Valverde, 1900, p.37).

Desde la perspectiva degeneracionista, estos síntomas eran evidencias del desequilibrio psíquico antes descrito, idea sobre la patogenia que tuvo una notable influencia en la mayoría de los autores. Así, Martínez Valverde (1900, p.200) escribía sobre los degenerados:

Fechos hombres y alcanzado todo su desarrollo son seres complejos y heterogéneos, desproporcionados en sus facultades psíquicas, con cualidades contradictorias, insuficientes para ciertas cosas, sobresalen en otras … Su sensibilidad es especial, pero en cambio falta más o menos el juicio, la rectitud y el sentido moral y sobre todo la perseverancia, la lógica y la unidad de dirección, así en lo intelectual como en los actos de su vida.

En la misma línea, Mateo Bonafonte (1899, p.18) definía al degenerado de acuerdo con estos parámetros: “Les falta la reflexión y la constancia de una dirección útil y personal, la lógica, la unidad de acción en el curso de sus determinaciones”.

Vemos cómo las diferentes formas de locura requerían un atento seguimiento de la evolución clínica, y, por tanto, resultó de gran importancia la identificación de los síntomas afectivos de los estadios iniciales de la enfermedad. Como ejemplo, Vicente Ots y Esquerdo (1903b, p.400) definía dos modelos diferenciados en el comienzo de la demencia precoz. Por un lado, describía un grupo de pacientes apáticos que “desde niños se les ve apartarse de sus compañeros, aislarse retraerse, sufrir frecuentes distracciones, ser apáticos, irresolutos, paréticos mentales” frente a otros enfermos denominados “expansivos”, que solían “expansionarse con el primer advenedizo, trabando íntima familiaridad; aprecian, juzgan y critican problemas que desconocen”. Por su parte, Enrique Fernández Sanz (1908) describía el comienzo de la demencia precoz subrayando las conductas menos aparentes del paciente descrito:

Al servir la mesa cometía extravagancias y simplezas, que en un principio fueron tomadas por bromas un poco necias (no servía vino a nadie, quitaba el pan etc.) … estaba triste, preocupado, experimentando ansiedad vaga, con imposibilidad de prestar atención a los menesteres de su oficio, y se hizo suspicaz, creyendo que te engañaban al darle la cuenta de su salario (Fernández Sanz, 1908, p.287).

Los grandes referentes de la psicopatología francesa del periodo de entresiglos – Séglas, Chaslin –, que fueron citados con asiduidad en las publicaciones españolas, subrayaron la importancia de la precisión en la descripción de las manifestaciones del enfermo. Dada la inespecificidad de los síntomas, más allá de buscar un elemento concreto diferenciador, la exploración psicopatológica consistiría en detectar las peculiaridades en la expresión de afectos, cogniciones y actitudes del paciente. A modo de ejemplo, Ots y Esquerdo, en un caso de estupor melancólico, diferenciaba el cuadro citado de otras entidades nosológicas como la demencia, ateniéndose a una matizada valoración de aspectos externos del comportamiento que permitían intuir la experiencia del paciente:

Aunque tanto en uno como en otro enajenado existe esa indiferencia apática; la que presenta el demente tiene por característico el no ser tan completa como en el estupor, puesto que en esta afección hay una abstracción tan absoluta en el enfermo que no puede la mayoría de las veces exteriorizar sus reacciones sensoriales y sensitivas; el estuporoso nada dice al ser impresionado desagradablemente, pero su actitud y la expresión de su mirada revelan claramente que no es indiferente a la excitación recibida (Ots y Esquerdo, 1893b, p.340).

Por su parte, Fernández Victorio y Cociña (1906, p.14) defendía su actitud antilocalizacionista, que “no admite que pueda localizarse la conciencia del yo, ni la facultad intelectiva, volición etc.”, y clarificaba que la base de la exploración residía en una observación atenta y en la interpretación precisa de los gestos, actitudes y contenidos mentales. Esta perspectiva resultaba clave en los diagnósticos diferenciales. Así, el mismo autor publicó un caso de demencia precoz heboidofrénica, donde sustentó el diagnóstico diferencial con una depresión melancólica de la siguiente forma:

La expresión del melancólico, así como su actitud, son de sufrimiento, de inquietud, de dolor moral … En nuestro enfermo la actitud es pasiva, más o menos fija, tendiendo a la espasticidad; la fisonomía inexpresiva, indiferente; el delirio poco acusado siempre y más bien polimorfo que de fondo depresivo; las reacciones en la actualidad escasas, pero en varias ocasiones violentas y agresivas y siempre bruscas o fugaces (Fernández Victorio y Cociña, 1908, p.41).

Estos diagnósticos diferenciales evidenciaban la necesidad de mantener una visión integrada de la clínica, ya que la percepción correcta de un síntoma exigía captar su relación con la experiencia global del paciente. De hecho, las descripciones clínicas mostraban un intento de exponer la psicopatología con una coherencia interna. Esta voluntad de describir el síntoma desde esta perspectiva llevaba a incluir de forma cada vez más frecuente material escrito, de forma más extensa de lo que solían consignarse en décadas anteriores y sin que la voluntad de justificar el diagnóstico fuese el interés exclusivo, sino que también reflejaba la importancia de representar la peculiaridad de la expresión clínica del caso, para lo que recurrían a textos mucho más extensos y variados. Hemos de recordar que el interés por la escritura de los pacientes con finalidad diagnóstica o terapéutica ya aparecía en autores tan representativos como Louis Victor Marcé y Brierre de Boismont en la psiquiatría francesa de mediados del siglo XIX. Rigoli (2001, p.245) señala su función esencial, dar un estatus clínico a la expresión de la locura y compensar las deficiencias de la descripción sintomática reflejando la realidad subjetiva del paciente y mostrando así su verdadera naturaleza, más allá de los signos evidentes. Igualmente, a principios del siglo XX, autores como Philippe Chaslin (1912) utilizaban los escritos de los pacientes para ilustrar las peculiaridades de la patología individual. También otros autores españoles, como Joaquín Gimeno Riera o José Salas y Vaca, director del Manicomio de Leganés en el primer cuarto del siglo XX, consideraban los textos de los pacientes como una ventana privilegiada a su mente y los utilizaban como ilustración y clarificación de su psicopatología (Villasante, 2018; Candela, Villasante, 2013). A modo de ejemplo, en el ya citado caso de locura religiosa descrito por Ots y Esquerdo (1892) se incluye una extensa copia de los escritos del paciente al Provincial de la Compañía de Jesús enunciándole los milagros del Padre que, según relata, le había salvado de la influencia del demonio, así como su testamento, donde muestra de forma detallada sus delirios místicos. Por su parte, Joaquín Gimeno Riera (1909) publicó, el mismo año en que el libro de Serieux y Capgras vio la luz, un caso diagnosticable como delirio de interpretación. En la introducción del artículo recordaba el hecho nuclear del cuadro, la interpretación sistemática, y su clínica, basada en la ausencia de alucinaciones y en la lucidez conservada de los pacientes, que se expresaba de forma evidente en sus textos escritos, lo que hacía que raramente fuesen tratados por especialistas. Para ejemplificar el caso, publicó un texto autobiográfico de un paciente ingresado en el Manicomio de Zaragoza, donde se detalla con excelente precisión la experiencia delirante y su evolución biográfica. Años más tarde, el mismo autor publicó un caso de alucinosis que diagnosticó referenciando a Chaslin y sus “tipos clínicos de espera”, ya que no respondía a ningún estereotipo clínico reconocible. Se trataba de un enfermo que sufría alucinaciones psíquicas de Baillarger, por las que llevaba 20 años ingresado en la institución que regentaba el autor, el Manicomio Provincial de Zaragoza. Para ilustrar el caso recogía tres cartas textuales donde el enfermo describió su experiencia alucinatoria, como víctima de fuerzas espirituales que establecían diálogos entre sí y con él; y donde precisaba los cambios que experimentó en distintos momentos de su vida gracias a un largo texto que el autor le demandó para explicar su caso (Gimeno Riera, 1912).

## Consideraciones finales

La psiquiatría española, dado lo tardío de su proceso de institucionalización, a partir de la segunda mitad del siglo XIX tuvo como objetivo inicial su asentamiento como una especialidad relevante dentro del campo de la medicina. Esta pretensión le llevó a defender postulados teóricos basados en el positivismo, oponiéndose a la escuela psicologicista (Novella, 2013a), y proponiendo una aproximación objetivante y fragmentaria del psiquismo. Los autores de la escuela catalana, encabezada por Juan Giné y Partagás, desarrollaron su modelo de acuerdo con un organicismo militante, donde la subjetividad del loco ocupaba un espacio limitado y confiaron en que el papel de la naciente especialidad se vería reforzado por el nuevo desarrollo científico, al que se confería un papel fundamental en la regeneración política y social del país.

A partir de la década de 1880 se publicaron los primeros trabajos influidos por la teoría de la degeneración en los que, progresivamente, se introdujo la nueva psicopatología francesa. La historiografía ha subrayado el papel de dicha teoría en la somatización de la enfermedad mental (Dowiggin, 1991; Pick, 1989) así como en la intervención social del psiquiatra a través de la extensión del concepto de locura a trastornos menores, facilitando la profesionalización y legitimación de su rama del saber a través de una patologización de la sociedad (Caponi, 2012). De hecho, la bibliografía existente insiste en considerar al periodo estudiado como una etapa de objetivación del paciente, considerando la aparición del psicoanálisis como un fenómeno aislado, sin continuidad con el desarrollo previo de la medicina mental. Sin embargo, el trabajo nos permite ver que, aunque la base teórica del degeneracionismo era fuertemente organicista y contenía aspectos rotundamente reduccionistas, simultáneamente a su aparición se produjo en España una modificación de la perspectiva clínica, ya que introdujo diversos cambios conceptuales que hicieron posible avanzar en un modelo que permitiera captar la experiencia interna de la locura. Tuvo lugar en un contexto cultural (expansión del espiritualismo psicológico en décadas previas, la difusión de la novela realista y naturalista y su nueva perspectiva del relato sobre el individuo) y teórico (la importación de una mirada subjetiva más profunda en torno a la psicopatología y la introducción de un modelo nosológico donde se integraba la perspectiva temporal de la enfermedad mental), que facilitaba una aproximación nueva a la locura y a la vivencia del individuo que la sufre.

Fue importante en este proceso la crisis finisecular de las instituciones psiquiátricas y el fracaso, veladamente reconocido, para encontrar una respuesta práctica al tratamiento de la locura (Plumed, Rojo, 2016), con lo que el papel del especialista desde la perspectiva clínica se centraba en el conocimiento de la enfermedad y sus sutilezas, necesario para discriminar entre locura y razón y, en el primer caso, sus distintas variedades. Con ello, nos encontramos relatos clínicos donde la perspectiva biográfica, la unidad y coherencia de la experiencia psíquica, así como los aspectos afectivos y volitivos menos aparentes cobraron mayor importancia y definieron una nueva visión del enfermar. Esto nos permite afirmar que, en las décadas previas a 1910, se había desarrollado una nueva cultura psiquiátrica, paralela a los discursos más reduccionistas, que facilitó la introducción de modelos teóricos que primaban el estudio de la interioridad, principalmente las ideas psicoanalíticas, que fueron, generalmente, valoradas de forma positiva por los autores más relevantes de los años 1920 en España (Carles et al., 2000). De la misma forma, la humanización del sujeto que propugnaba esta perspectiva debió influir en la crítica a la pobrísima estructura asistencial de la época, que fue constante durante las décadas posteriores (Huertas, 2002, p.177-178).

## Data Availability

No están en repositorio.
